# Genome-Wide Identification and Expression Analysis of the PEPC Gene Family in *Zanthoxylum armatum* Reveals Potential Roles in Environmental Adaptation

**DOI:** 10.3390/biology14111605

**Published:** 2025-11-16

**Authors:** Ruxin Xu, Huamin Liu, Chongyu Liu, Maoqin Xia, Dalan Feng, Yongxing Zhu, Chong Sun, Xia Liu, Mi Kuang, Xia Gong, Zheng Chen, Shanrong Li, Zexiong Chen

**Affiliations:** 1 Chongqing Key Laboratory of Economic Plant Biotechnology, Collaborative Innovation Center of Special Plant Industry in Chongqing, College of Smart Agriculture, Chongqing University of Arts and Sciences, Chongqing 402160, China; 18069222925@163.com (R.X.); liuhuanin@126.com (H.L.); ayjob2000@163.com (C.L.); xiamq@cqwu.edu.cn (M.X.); zbgqsc1987@163.com (C.S.); 2 Chongqing Key Laboratory of Forest Ecological Restoration and Utilization in the Three Gorges Reservoir Area, Chongqing Academy of Forestry Sciences, Chongqing 400036, China; fengdalang@163.com; 3 Hubei Key Laboratory of Spices & Horticultural Plant Germplasm Innovation & Utilization, Yangtze University, Jingzhou 434023, China; xbnlzyx@163.com; 4 Chongqing Agricultural Technology Promotion General Station, Chongqing 401120, China; kim437@126.com (M.K.); goldcherry@163.com (S.L.); 5 Sichuan Provincial Institute of Agricultural Special Plants, Neijiang 641200, China; gongxiavip858@163.com (X.G.); 19941953@163.com (Z.C.)

**Keywords:** *Zanthoxylum armatum*, phylogenetic analysis, woody plants, stress response, expression pattern

## Abstract

Phosphoenolpyruvate carboxylase (PEPC) is a crucial enzyme in plant photosynthesis and stress responses, yet its gene family remained uncharacterized in *Zanthoxylum armatum*. This study presents the first genome-wide identification and comprehensive analysis of the PEPC gene family in *Z. armatum*. A total of 12 *ZaPEPC* genes were identified and classified into plant-type (PTPC) and bacterial-type (BTPC) subfamilies based on phylogenetic analysis. These genes exhibited conserved protein domains but distinct gene structures, with evidence of gene duplication events contributing to family expansion. Promoter analysis revealed an abundance of stress- and hormone-responsive *cis*-elements, particularly those related to light, abscisic acid (ABA), and methyl jasmonate (MeJA). Expression profiling demonstrated that *ZaPEPC* genes display environment-specific expression patterns, with *ZaPEPC7* and *ZaPEPC11* showing significantly higher expression in high-altitude, high-light environments (Yunnan) compared to other regions (Shandong and Chongqing). Co-expression network analysis further indicated interactions between specific *ZaPEPCs* and stress-related transcription factors. These findings systematically reveal the molecular characteristics and potential roles of the *ZaPEPC* gene family in environmental adaptation, providing valuable genetic resources and a theoretical foundation for improving stress tolerance and photosynthetic efficiency in *Z. armatum* through molecular breeding.

## Simple Summary

This study presents the first comprehensive analysis of the PEPC (Phosphoenolpyruvate carboxylase) gene family in the Sichuan pepper plant (*Zanthoxylum armatum*). PEPC, a key enzyme in photosynthetic carbon fixation and stress response, was investigated using integrated bioinformatics and molecular approaches. Twelve *PEPC* genes were identified, exhibiting distinct expression patterns across environmental gradients. Notably, the highest expression levels were observed in high-altitude, high-light regions of Yunnan, China. This finding indicate that these genes may help *Z. armatum* adapt to diverse climates and resist stresses such as drought. This finding provides genetic resources and a theoretical basis for improving photosynthetic efficiency, stress response, and fruit quality in *Z. armatum*, offering important value for promoting the breeding and sustainable development of this economically significant crop.

## 1. Introduction

*Zanthoxylum armatum* DC. commonly known as Sichuan pepper, is an important characteristic plant in China with both medicinal and edible applications. It plays an increasingly vital role in regional economic development and rural revitalization [1]. China is rich in *Z. armatum* resources, with its cultivation area expanding at an annual rate of 20–30% and an annual output exceeding one million metric tons [2]. Owing to its unique numbing and aromatic flavor, along with a variety of bioactive compounds, it enjoys considerable market popularity. This crop not only generates substantial economic benefits for farmers in mountainous regions but also holds broad application potential in the food, spice, and traditional Chinese medicine industries [3]. However, climate change-induced abiotic stressors, such as extreme heat and drought, are increasingly threatening global agricultural productivity. These challenges not only jeopardize the yield and quality of staple crops like wheat [4] but also pose serious threats to the sustainable cultivation of many high-value cash crops. As a representative high-value cash crop, *Z. armatum* faces significant industrial challenges. Its cultivation is mainly concentrated in topographically complex mountainous and hilly areas, where it is often exposed to multiple abiotic stresses, including seasonal drought, poor soil conditions, high temperature and intense light, as well as low temperature and frost [5,6,7,8]. These factors severely constrain its growth and development, the accumulation of secondary metabolites, and ultimately the improvement of both yield and quality. Therefore, a deeper understanding of the molecular regulatory networks underlying the environmental stress responses of *Z. armatum* is crucial for breeding stress-resistant varieties and ensuring the stability and sustainable development of this industry.

During long-term evolution, plants have developed complex and sophisticated molecular mechanism to cope with environmental stresses [9]. These mechanisms involve the coordinated regulation of multiple key gene families, which enhance a plant’s adaptive capacity by modulating its physiological and biochemical processes. For example, in wheat, the BAG co-chaperone family has been shown to play a central role in mediating responses to both heat and cold stress through its molecular chaperone activity [10]. Among these regulatory mechanisms, phosphoenolpyruvate carboxylase (PEPC), a key node enzyme linking primary carbon and nitrogen metabolism, has functions extending far beyond its initially recognized role in photosynthesis in C4 and crassulacean acid metabolism (CAM) plants [11,12]. In C3 plants, including woody species, PEPC catalyzes the irreversible carboxylation of phosphoenolpyruvate (PEP) to generate oxaloacetate (OAA). This reaction provides substrates for replenishing carbon skeletons in the tricarboxylic acid (TCA) cycle, supports the synthesis of amino acids such as aspartate, and helps maintain intracellular pH homeostasis [13,14]. A growing body of evidence indicates that PEPC plays a central role in plant responses to various abiotic and biotic stresses, including drought, salinity, low temperature, nutrient deficiency, and pests and diseases [15,16,17,18,19]. For instance, under stress conditions, the upregulation of PEPC activity enhances the synthesis of organic acids, thereby contributing to ion balance and redox homeostasis. Concurrently, its carbon fixation function supports the reallocation of energy and carbon skeletons necessary for effective stress responses [11,20,21].

The PEPC enzyme is encoded by a multigene family. In model species such as *Arabidopsis thaliana* [22], *Oryza sativa* (rice) [23], and *Setaria italica* (foxtail millet) [24], the PEPC gene family members have been systematically identified and classified into distinct subfamilies—primarily plant-type (PTPC) and bacterial-type (BTPC)—based on gene structure and phylogenetic relationships [16,21,25]. These members exhibit significant functional diversification: some perform essential housekeeping roles, while others are specifically induced to participate in stress response processes [13,16,22,23]. Plant-type PTPCs are homotetramers composed of approximately 100–110 kDa polypeptides. Their encoding genes typically contain 9–10 introns. These PEPCs are characterized by a conserved serine residue at the N-terminus and a distinctive C-terminal QNTG (Gln-Asn-Thr-Gly) tetrapeptide motif. In contrast, bacterial-type BTPCs form heterooctamers consisting of 116–118 kDa polypeptides. The genes encoding BTPCs possess a greater number of introns (19–21), and the resulting proteins lack the N-terminal serine, instead featuring a C-terminal (R/K) NTG (Arg/Lys-Asn-Thr-Gly) tetrapeptide [13,23]. The presence of the N-terminal serine is a hallmark of plant-type PEPCs, allowing reversible phosphorylation by PEPC kinase—a key regulatory mechanism absent in bacterial-type enzymes [26]. This post-translational modification not only governs primary carbon fixation in C4 and CAM plants but is also widely involved in carbon metabolism regulation in non-photosynthetic tissues, playing critical roles in processes such as seed germination and storage compound synthesis in cereals [27]. Bacterial-type PEPCs, on the other hand, primarily function to replenish tricarboxylic acid cycle intermediates and recapture CO_2_ released during respiration [14,23]. Beyond these hallmark N-terminal and C-terminal signatures, comparative analyses of PEPC proteins across diverse plant species have revealed a set of conserved protein motifs that are critical for their structure and function. For instance, plant-type PTPCs typically contain a set of conserved motifs that are stable in number (e.g., up to ten) and exhibit a conserved sequential order, whereas bacterial-type BTPCs, while also harboring these core motifs, may display alterations in their arrangement or the absence of specific individual motifs. Differences in the composition and configuration of these motifs serve not only as molecular hallmarks for distinguishing the PTPC and BTPC subfamilies but also suggest potential functional divergence between the two subfamilies over the course of long-term evolution.

The structural and regulatory distinctions between the two PEPC types underlie a clear division of labor and functional synergy in planta. Substantial evidence confirms that specific *PEPC* family members are selectively induced during plant stress responses, a pivotal function demonstrated across diverse species. For instance, in *A. thaliana*, three of its four *PEPC* genes belong to the plant-type subfamily, while the remaining one is a bacterial-type PEPC. A bacterial-type PEPC has likewise been identified in rice [23]. The functional significance of this gene family in stress tolerance is increasingly clear. In soybean, among its 10 *GmPEPC* genes, *GmPEPC6*, *GmPEPC8*, and *GmPEPC9* are notably induced by aluminum toxicity, chilling, and salt stress [16]. Similarly, *AtPPC4* in *Arabidopsis* is activated under drought stress [22]. Such abiotic adversities, including drought, salinity, and cold, are widely reported to strongly induce the expression of specific *PEPC* genes [22,28]. The observed decline in PEPC activity during drought stress correlates with reduced photosynthetic and electron transport rates, suggesting that PEPC contributes to plant drought resistance, potentially through the regulation of photosynthesis [29]. However, compared with annual herbaceous plants, our knowledge of the *PEPC* gene family in perennial woody plants remains limited, particularly for economically significant specialty tree species like *Z. armatum*. Key aspects such as gene family composition, expression regulation, and functional divergence are still poorly characterized. Given the long life cycle and complex habitats of *Z. armatum*, it is an open and pressing scientific question whether its PEPC gene family has evolved unique regulatory mechanisms to adapt to variable environments and recurrent stresses.

The rapid advancement of high-throughput sequencing technologies in recent years has enabled whole-genome sequencing for a vast number of plant species, creating unprecedented opportunities for genome-wide identification and analysis of gene families. By integrating multi-omics approaches, such as genomics, transcriptomics, and proteomics, it is now feasible to comprehensively identify members of specific gene families, elucidate their evolutionary relationships, gene structures, conserved motifs, and promoter *cis*-regulatory elements. When coupled with expression profiling, these strategies can effectively reveal the potential functions of gene family members in plant development and stress responses [10,30]. This genome-scale analytical framework has thus become a powerful tool for mining key stress-resistance genes and clarifying the molecular basis of plant environmental adaptation. In evolutionary genomics, syntenic blocks represent conserved ancestral genomic segments. Analyzing synteny across species provides insights into the architecture of ancestral genomes and elucidates the evolutionary forces, such as polyploidization and chromosomal rearrangements, that have driven genome diversification following species divergence [31].

The recent publication of a high-quality reference genome for *Zanthoxylum armatum* [32] provides an ideal foundation for such an investigation. As an autotetraploid species (2n = 4x = 132) with a contig N50 of 3.31 Mb, this genome assembly offers a continuous and reliable framework for accurate gene annotation and family-wide analysis. Therefore. using integrated bioinformatics approaches, we performed comprehensive analyses of their phylogenetic relationships, chromosomal locations, gene structures, conserved protein domains, physicochemical properties, and promoter *cis*-regulatory elements. Furthermore, by leveraging transcriptome data from *Z. armatum* under diverse abiotic stresses, we investigated the expression patterns of *ZaPEPC* genes. Our work aims to uncover the molecular characteristics and functional divergence of the PEPC gene family in this species, clarify its potential roles in environmental adaptation and abiotic stress responses, and provide important theoretical insights and genetic resources for further elucidating stress resistance mechanisms in woody plants. Ultimately, these findings will support molecular-assisted breeding efforts aimed at developing new, stress-resistant cultivars of *Z. armatum*.

## 2. Materials and Methods

### 2.1. Plant Materials

Leaf samples from three-year-old *Z. armatum* plants in the common garden at Chongqing University of Arts and Sciences. These plants were originally from Shandong Province (SD; 34°58′59″ N, 117°17′41″ E), Chongqing City (CQ; 29°12′50″ N, 105°50′47″ E), and Yunnan Province (YN; 25°39′54″ N, 101°54′25″ E), China. From each plant, we selected current-year shoots and collected the third compound leaf from the apex to ensure sample uniformity. Three biological replicates were established for each regional sample. After collection, the leaves were immediately put into liquid nitrogen for quick freezing treatment, and stored in ultra-low temperature freezing equipment (−80 °C) for RNA extraction. Detailed collection and processing of research materials can also be found in our previous published paper [33,34], the primary transcriptome data have been deposited in the National Genomics Data Center with the primary accession number PRJCA037036.

### 2.2. Identification of PEPC Gene Family Members

To identify the *PEPC* gene family in *Z. armatum*, we employed two independent searches against its published genome [32]. First, we used a Hidden Markov Model (PF00311) in the Pfam database and used HMMER3.0 software to identify the *PEPC* genes of *Z. armatum* with an E value < 10^−40^ as the threshold. The second approach involved retrieving the protein sequences of previously reported *PEPC* gene family members from *Arabidopsis* official website TAIR (https://www.arabidopsis.org) (accessed on 15 January 2024). A BLASTP search was then performed on NCBI website (the National Center for Biotechnology Information Website: https://www.ncbi.nlm.nih.gov) (accessed on 15 January 2024) using these sequences as queries, with an E-value threshold set to 10^−5^, to obtain the FASTA formats of both the protein and gene sequences of the candidate *ZaPEPC* genes. The candidate genes common to both searches were consolidated. Subsequently, the presence of a complete PEPCase domain in each candidate was verified via the conserved domain database (CDD) at NCBI (https://www.ncbi.nlm.nih.gov/Structure/cdd/wrpsb.cgi) (accessed on 15 January 2024). Only sequences possessing the full domain were retained for further analysis.

### 2.3. Physicochemical Properties and Subcellular Localization Analysis of ZaPEPC Gene Family

Isoelectric points, molecular weights, sequence lengths, instability indices, lipid coefficients, and average hydrophilicity coefficients were calculated for the ZaPEPC proteins using ExPASy (https://web.expasy.org/protparam/) (accessed on 20 January 2024), and their subcellular localization was predicted using Plant-mPLoc (http://www.csbio.sjtu.edu.cn/bioinf/plant-multi/) (accessed on 20 January 2024).

### 2.4. Phylogenetic Analysis of the PEPC Gene Family and Collinearity Analysis of ZaPEPC Genes with Other Species

To elucidate the evolutionary relationships of *PEPC* genes, the PEPC protein sequences of *Arabidopsis thaliana* (At), *Oryza sativa* (Os), *Zea mays* (Zm), *Glycine max* (Gm), *Setaria italica* (Si) and *Zanthoxylum armatum* (Za) were subjected to multiple sequence comparisons using MAFFT v7.0 [35], and subsequently, poorly conserved and gap-rich regions were trimmed from the alignment to generate a high-quality multiple sequence alignment for robust phylogenetic analysis. MEGA v12.0 software [36] was used to construct a multi-species phylogenetic tree using the neighbor-joining (NJ) method; the reliability of the tree topology was evaluated by bootstrap analysis with 1000 replicates and all other parameters were set as default values. Cereal protein sequences were obtained from the phytozome database (https://phytozome.jgi.doe.gov/) (accessed on 22 January 2024). The details for the remaining four crops were obtained from the maize database (https://www.soybase.org/) (accessed on 22 January 2024), the rice database (https://www.soybase.org/) (accessed on 22 January 2024), the soybean database (https://www.soybase.org/) (accessed on 24 January 2024), and the *A. thaliana* database (http://www.arabidopsis.org) (accessed on 22 January 2024).

For collinearity analysis, nucleic acid sequences and annotation files from the following public databases: *Arabidopsis thaliana* (TAIR; https://www.arabidopsis.org) (accessed on 25 January 2024), *Oryza sativa* (the Rice Annotation Project Database; https://rapdb.dna.affrc.go.jp) (accessed on 25 January 2024), *Zea mays* (MaizeGDB; https://www.maizegdb.org) (accessed on 27 January 2024), *Glycine max* (SoyBase; https://www.soybase.org) (accessed on 27 January 2024), and Setaria *italica* (phytozome; https://phytozome.jgi.doe.gov/) (accessed on 28 January 2024). These files were used as input for the “One Step MCScanX” tool in TBtools v2.056 [37]. The tool first performed an all-against-all BLASTP analysis (E-value cutoff ≤ 1×10^−10^) to identify homologous genes, and then the MCScanX algorithm was applied to detect collinear genomic blocks between *Z. armatum* and each reference genome. The syntenic blocks containing *PEPC* genes were highlighted.

### 2.5. Analysis of ZaPEPC Gene Structure and Conserved Motifs and Promoter Cis-Regulatory Elements

The GFF file of *Z. armatum* gene annotation was searched using TBtools v2.056, and the intron and exon structures of each member of the *ZaPEPC* family were visually analyzed. The online tool MEME v4.12.0 (http://memesuite.org/tools/meme) (accessed on 2 February 2025) was used to analyze the conserved protein motifs of *PEPC* family members in Z. *armatum*, and the motif search value was set to 10 motifs. According to the whole genome data of Z. *armatum* [32], the sequence of 2000 bp upstream of the start codon (ATG) was extracted as the candidate promoter sequence, and the *cis*-acting element analysis was performed using the PlantCARE (http://bioinformatics.psb.ugent.be/webtools/plantcare/html/) (accessed on 2 February 2025) website. Finally, TBtools v2.056 were used for visual mapping.

### 2.6. Chromosomal Localization and Gene Duplication Analysis

The chromosomal locations of the *ZaPEPC* genes (including flanking genes) and the genome-wide gene density were visualized using TBtools v2.056. Gene density visualization was performed as follows: Firstly, gene location visualize from GFF/GTF function was utilized, with the complete genome annotation file (in GFF/GTF format) used as input. Subsequently, the draw gene density module was selected. This function employs a sliding window algorithm to calculate the number of genes per megabase (Mb) along each chromosome. Additionally, the Simple Ka/Ks Calculator in TBtools v2.056 was used to analyze the selection pressure on duplicated gene pairs. The analysis required three input files: the CDS sequences, protein sequences of *Z. armatum*, and a list of collinear *ZaPEPC* gene pairs. The program output the non-synonymous (Ka) and synonymous (Ks) substitution rates and their ratio (Ka/Ks) to determine the selection pressure after gene replication.

### 2.7. Analysis of ZaPEPC Gene Expression Pattern in Z. armatum with Different Latitudes

Transcriptome sequencing was performed following the well-established protocol detailed in our previous publications [33,34]. The expression characteristics of the *PEPC* gene family were analyzed by transcriptome sequencing of leaf samples collected from three-year-old *Z. armatum* plant from three different regions: Shandong (SD), Chongqing (CQ), and Yunnan (YN) in China. For each region, three biological replicates were sequenced. The transcriptome sequencing was performed using Illumina and compared with the reference genome (*Z. armatum*_genome fa.gz). After obtaining the original data, low-quality reads were eliminated, HISAT2 v2.2.2.0 software was used for annotation, and the reads mapped to each gene were analyzed using the feature counting program to calculate the number of reads per thousand base fragments (FPKM) value of each million mapping reads of the gene. Finally, the FPKM values of *PEPC* gene family genes in *Z. armatum* were extracted and an expression clustering heat map was drawn using TBtools v2.056.

### 2.8. RNA Extraction and RT-qPCR Expression Analysis

To validate the transcriptome data, RT-qPCR was performed using the same RNA samples described in Section 2.7. Total RNA was extracted from *Z. armatum* leaves using an RNA extraction kit (Beijing Tiangen Biochemical Technology Co., Ltd., Beijing, China, DP220706). The RNA concentration was measured using a NanoDrop 2000 spectrophotometer (Thermo Fisher Scientific, Waltham, MA, USA). cDNA was generated by reverse transcription using the HiScript III 1st Strand cDNA Synthesis Kit (+gDNA wiper) (Nanjing Vazyme Biotech Co., Ltd, Nanjing, China). For RT-qPCR, a CFX Connect Real-Time Fluorescent Quantitative PCR Detection System (Bio-Rad, Hercules, CA, USA) was utilized. The RT-qPCR was conducted in a 25 µL reaction mixture, which included 12.5 µL of enzyme, 1 µL forward primer (10 µmol/L), 1 µL reverse primer (10 µmol/L), 1 µL cDNA template, and 9.5 µL ddH_2_O. The PCR protocol consisted of an initial pre-denaturation at 94 °C for 20 s, followed by 35 cycles of denaturation at 94 °C for 10 s, annealing at 60 °C for 20 s, and extension at 72 °C for 10 s. The relative expression levels of the candidate genes across different samples were determined using the 2^−ΔΔCT^ method. The *ZaUBQ* gene was used as an internal control for normalization, and three replicates were analyzed for each sample [38]. The expression data were then analyzed using SPSS v20.0 to assess the significance of the differences. Primers were designed using Primer v5.0 software and synthesized by Shanghai Sangon Biological Engineering Co., Ltd., Shanghai, China. The sequences of all primers used for RT-qPCR are listed in Supplementary Materials (Table S1).

### 2.9. Co-Expression Relationship Network and Kyoto Encyclopedia of Genes and Genomes (KEGG) Analysis of ZaPEPC Genes

To explore the regulatory networks of the *ZaPEPC* genes, a co-expression analysis was performed using transcriptome data (FPKM) from *Z. armatum* leaves sampled from three latitudinal regions with three biological replicates each. Genes with low expression (FPKM < 1 in all samples) were first filtered out. Subsequently, the Pearson Correlation Coefficient (R) between each *ZaPEPC* gene and all other genes was calculated using the R rsgcc package v4.5.0 [39]. Gene pairs with an absolute R value ≥ 0.98 and a statistically significant *p*-value (*p* ≤ 0.05) were identified as significantly co-expressed, yielding a high-confidence expression matrix of 3746 genes for subsequent analysis. Finally, a co-expression network was visualized with Cytoscape v3.5.1 [40]. All genes significantly co-expressed with *ZaPEPCs* were compiled and subjected to KEGG pathway enrichment analysis in TBtools v2.056 [41], with a significance threshold of *p*-value < 0.05.

### 2.10. Statistical Analysis

Statistical significance (* *p* ≤ 0.05, ** *p* ≤ 0.01, *** *p* ≤ 0.001, **** *p* ≤ 0.0001) was assessed by Student’s *t*-test and one-way ANOVA. Data are expressed as mean ± SD. Analyses were performed using GraphPad Prism v9. In the co-expression network, gene pairs with a Pearson correlation coefficient |*R*| > 0.98 and a *p*-value ≤ 0.05 were deemed significant.

## 3. Results

### 3.1. Identification Analysis of PEPC Gene Members in Z. armatum

Twelve *PEPC* family members were identified in *Z. armatum* and named as *ZaPEPC1-ZaPEPC12* according to their physical locations (Table 1 and Table S2). Analysis of the candidate ZaPEPC protein parameters showed that the number of amino acids encoded by ZaPEPC members was between 867 and 1115. The molecular weight of the members ranged from 99.63 kDa to 127.93 kDa, the isoelectric point of the protein ranged from 5.78 to 7.33, the instability index ranged from 44.15 to 51.69, the aliphatic index ranged from 88.3 to 94.1, and the average hydrophobic index ranged from −0.412 to −0.307 (Table 1), indicating that all ZaPEPC proteins are hydrophilic. Analysis of secondary metabolic structures of the PEPC proteins indicates that the proportion of α-helix of *ZaPEPC* members was 56.09–61.87%, and the proportion of random coil was 27.86–33.5% (Table 2), indicating that α-helix and random coil played a major role in the formation of tertiary structure.

### 3.2. Phylogenetic Relationship, Analysis of Domains and Conserved Motifs of ZaPEPC Gene Family Members

**Figure 1:**
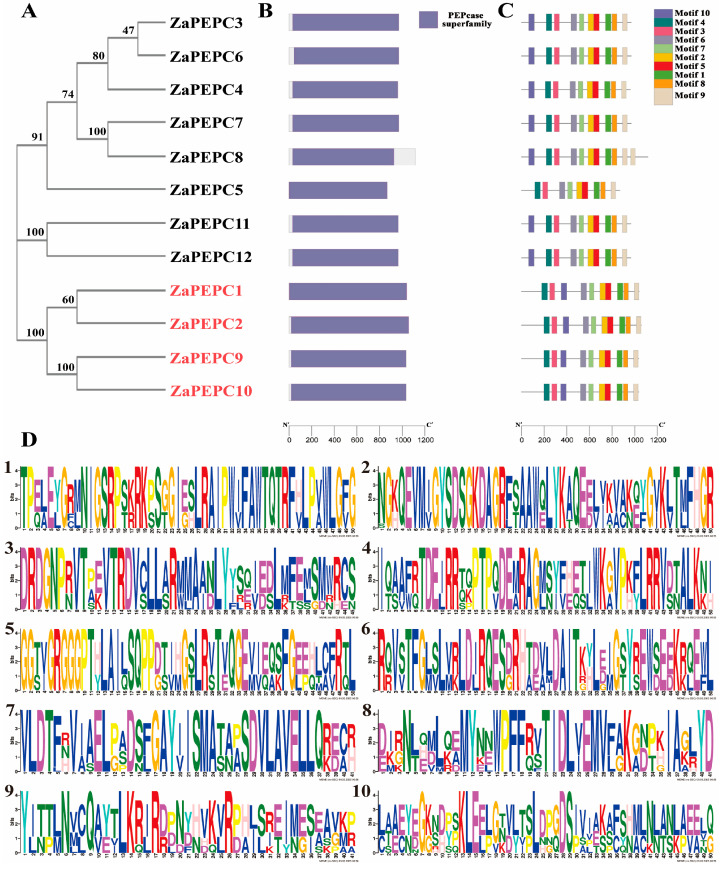
Phylogenetic relationship, conserved motif, and gene structure of the *PEPC* gene in *Zanthoxylum armatum*. (**A**) A neighbor-joining phylogenetic tree of ZaPEPC proteins. The tree was constructed based on multiple sequence alignments, and its reliability was assessed by bootstrap analysis with 1000 replicates. Numbers at the branches represent bootstrap support values (%). *ZaPEPC1*, *2*, *9*, *10* are bacterial-type BTPCs. The rest are plant-type PTPCs. (**B**) The conserved domains of ZaPEPC proteins. (**C**) Distribution of ten conserved protein motifs: different color blocks represent different conserved motifs. (**D**) Amino acid sequences of the conserved motifs (Motif 1–10): the sequence logo for each motif is shown, where the height of letters indicates the degree of amino acid conservation at each position, while the relative size of letters within the stack represents the frequency of each amino acid.

To elucidate the evolutionary relationships among the ZaPEPC proteins, a phylogenetic tree was constructed using the neighbor-joining method with 1000 bootstrap replicates. Phylogenetic classification of the 12 ZaPEPC members revealed two subfamilies: the bacterial-type (BTPC: *ZaPEPC1*, *2*, *9*, *10*) and the plant-type (PTPC: the remaining eight members) (Figure 1A). This evolutionary divergence was further reflected in their protein motifs. We firstly examined the conserved protein domains to establish the fundamental functional units. Conserved domain analysis confirmed that all ZaPEPC proteins possess complete PEPCase domains (Figure 1B). We identified finer sequence features by analyzing conserved motifs using MEME, which revealed ten predominant motifs (Figure 1C,D). *ZaPEPC* members contain 10 conserved motifs except for PEPC5. Moreover, except for PEPC5, which was missing motif10, the position and sequence of plant-type PEPC (PTPC) were similar, both containing motif1–10. However, the order of the bacterial BTPC and plant PTPC domains differed slightly, which manifested in the fact that motif10 was located in the third position in bacterial PEPC and the first position in plant PEPC (Figure 1C). This indicates that differences in these conserved motifs may affect the function of *ZaPEPCs*. To further characterize their functional motifs, we performed multiple sequence alignment (Supplementary Figure S1). This analysis revealed that all plant-type PTPCs possess a conserved serine residue at the N-terminus and a characteristic C-terminal QNTG (Gln-Asn-Thr-Gly) tetrapeptide. In contrast, all bacterial-type BTPCs lack the N-terminal serine and instead feature an (R/K) NTG (Arg/Lys-Asn-Thr-Gly) tetrapeptide at their C-terminus.

### 3.3. Chromosomal Localization, Gene Structure and Collinearity Analysis of ZaPEPC Gene

**Figure 2:**
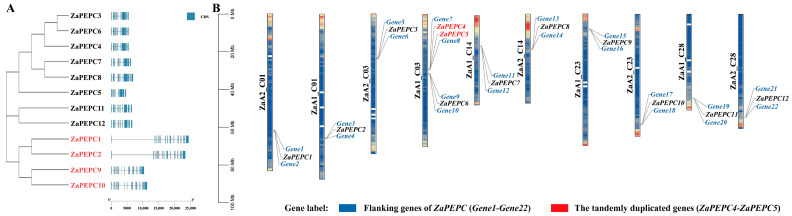
Gene structure and chromosomal distribution of *ZaPEPC* genes. (**A**) The structure of the *PEPC* gene: exon–intron structure of the *ZaPEPC* genes, where the blue box represents the exon and the white line connecting the exons represents the intron. (**B**) Chromosome distribution of the *ZaPEPC* gene family. The 12 *ZaPEPC* genes are unevenly distributed across five chromosomes of *Z. armatum*. To elucidate the duplication mechanisms of the *ZaPEPC1–12* genes, their physically flanking genes (genes 1–22) are displayed in blue, while the tandemly duplicated genes were labeled in red (for sequences IDs, please refer to the Table S3). The color gradient in the background of each chromosome represents the gene density, transitioning from blue (low gene density) to red (high gene density). The scale on the left is in mega bases (Mb).

To elucidate the genomic organization of the *ZaPEPC* gene family, we integrated an analysis of their chromosomal locations with their exon–intron structures (Figure 2). Members of the plant-type (PTPC) subfamily, including *ZaPEPC3*, *ZaPEPC4*, *ZaPEPC6*, *ZaPEPC7*, *ZaPEPC11*, and *ZaPEPC12* exhibited a relatively compact structure with 9–10 exons. In contrast, genes belonging to the bacterial-type (BTPC) subfamily (*ZaPEPC1*, *ZaPEPC2*, *ZaPEPC9*, and *ZaPEPC10*) possessed a more complex architecture characterized by 19–20 exons (Figure 2A). This pronounced divergence in gene structure underscores distinct evolutionary trajectories for the PTPC and BTPC subfamilies.

This structural divergence was further reflected in their genomic organization across the genome. The *12 ZaPEPC* genes were unevenly distributed across five chromosomes of *Z. armatum* (Figure 2B). A notable observation was the physical adjacency of *ZaPEPC4* and *ZaPEPC5* on chromosome 3 (their distance was 48,722 bp), forming a typical tandem gene cluster, which suggests a recent gene duplication event.

**Figure 3:**
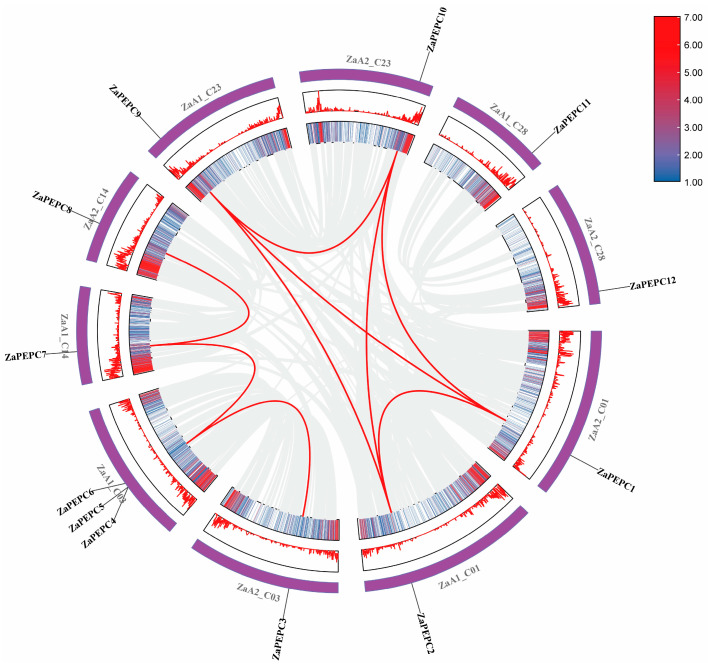
The distribution and synteny of the *ZaPEPC* gene family and gene replication events. From outer to inner: the outermost circle shows the physical location of the 12 *ZaPEPC* genes on their respective chromosomes; the middle circle displays the chromosome-wide gene density (blue-to-red gradient); the innermost part shows syntenic connections between duplicated gene pairs, suggesting recent gene duplication events.

To study the evolutionary process of the *PEPC* family genes in *Z. armatum*, we analyzed the collinearity of the *PEPC* gene family. We found that eight pairs of *ZaPEPC* genes were collinear, and *ZaPEPC4* and *ZaPEPC5* were tandem gene clusters on CHR3 (Figure 3). Among them, *ZaPEPC1*, *ZaPEPC2*, *ZaPEPC9*, and *ZaPEPC10* showed collinear relationships with each other. *ZaPEPC4* was collinear with *ZaPEPC7*, and *ZaPEPC3*, *ZaPEPC7*, and *ZaPEPC8* were also collinear. These findings indicate that the expansion of the ZaPEPC gene family was driven by multiple gene duplication events, including both tandem (e.g., *ZaPEPC4* and *ZaPEPC5*) and segmental (e.g., *ZaPEPC1* and *ZaPEPC2*) duplications, illustrating its complex evolutionary history.

The evolutionary selection pressure analysis showed that the Ka/Ks ratios of the nine gene pairs were greater than zero and lower than one (Table 3), indicating that they have undergone strong purifying selection. This evolutionary pressure preserves essential functional domains and implies that these genes have maintained their ancestral biological functions. A few gene pairs (*ZaPEPC1* and *ZaPEPC2*, *ZaPEPC7* and *ZaPEPC8*) showed a trend of close to neutral evolution, which may indicate certain functional flexibility or adaptability.

### 3.4. The Multi-Species Phylogenetic Analysis of PEPC Genes

**Figure 4:**
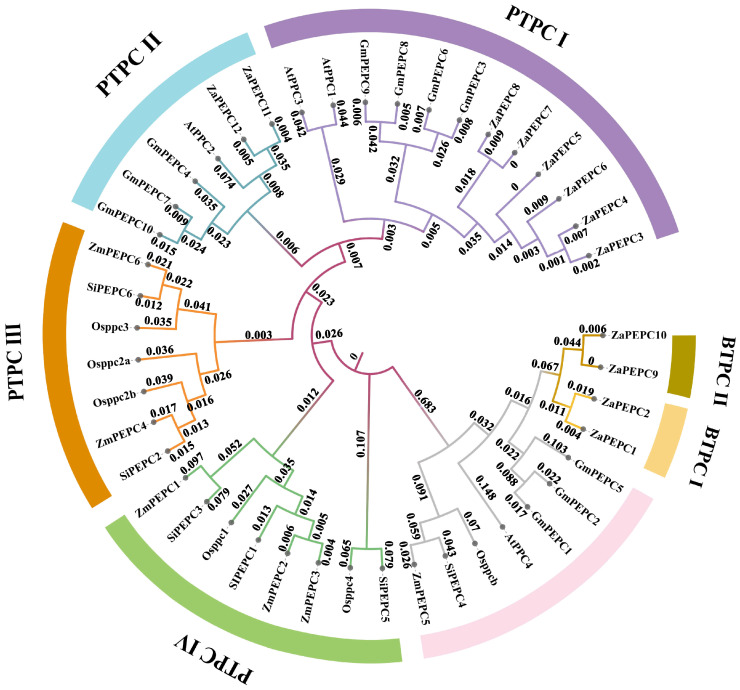
Phylogenetic analysis of PEPC proteins in soybean and other plant species. A neighbor-joining tree was constructed using 44 PEPC protein sequences from six species: four *AtPEPC* genes (*Arabidopsis thaliana*), six *OsPEPC* genes (*Oryza sativa*), six *ZmPEPC* genes (*Zea mays*), six *SiPEPC* genes (*Setaria italica*), six *GmPEPC* genes (*Glycine max*), and five *ZaPEPC* genes. The tree robustly segregates all PEPCs into two major clades: the plant-type PTPCs and the bacterial-type BTPCs. The PTPC clade is further subdivided into four groups (PTPC I-IV), while the BTPC clade splits into two (BTPC I–II). Branches with different colors represent different subgroups (PTPCI-PTPC IV; BTPCI-BTPC II) and numbers on the branch represents the genetic distance, which are in the units of the number of amino acid substitutions per site.

To study the evolutionary relationship between the ZaPEPC protein and other known species, we constructed a neighbor-joining (NJ) phylogenetic tree using 44 PEPC proteins from 6 different plant species, including 4 from *A. thaliana*, 6 from *Oryza sativa*, 6 from *Zea mays*, 10 from *Glycine max*, and 6 from *Setaria italica* (Table S2). The phylogenetic tree (Figure 4) shows that these PEPC-containing *ZaPEPC* genes could be divided into two subfamilies (PTPC and BTPC) based on their sequence similarity. The PTPC subfamily contained 33 members that were further divided into four groups (PTPC I–IV). The BTPC subfamily was further divided into two groups (BTPC I and BTPC II). Twelve *ZaPEPCs* were assigned to the following groups with high bootstrap values: *ZaPEPC3*, *ZaPEPC4*, *ZaPEPC6*, *ZaPEPC5*, *ZaPEPC7*, *ZaPEPC8* in the PTPC I group. *ZaPEPC11* and *ZaPEPC12* were included in the PTPC II group. *ZaPEPC1* and *ZaPEPC2* were also identified in the BTPC I group. *ZaPEPC9* and *ZaPEPC10* were included in BTPC II group (Table S4). Similar distribution patterns of PEPC components were found in *G. max* (Table S5). Phylogenetic analysis also revealed that the PTPC I and PTPC II family members of *Z. armatum* were closer to *GmPEPC3*, *GmPEPC6*, *GmPEPC8*, *GmPEPC9*, *GmPEPC4*, *GmPEPC7*, and *GmPEPC10* in the soybean homologous series. In addition, the bacterial-type PEPC of *Z. armatum* was closer to the homologous series of soybeans. This suggests that a set of ancestral PEPCs existed prior to the divergence of soybean and *Z. armatum*. This close phylogenetic relationship with soybean *PEPC*s, particularly within specific subfamilies, provides crucial evolutionary context and supports the use of soybean as a model for inferring the potential functions of the newly identified *ZaPEPC* genes.

### 3.5. Multi-Species Collinearity Analysis of PEPC Gene Family

**Figure 5:**
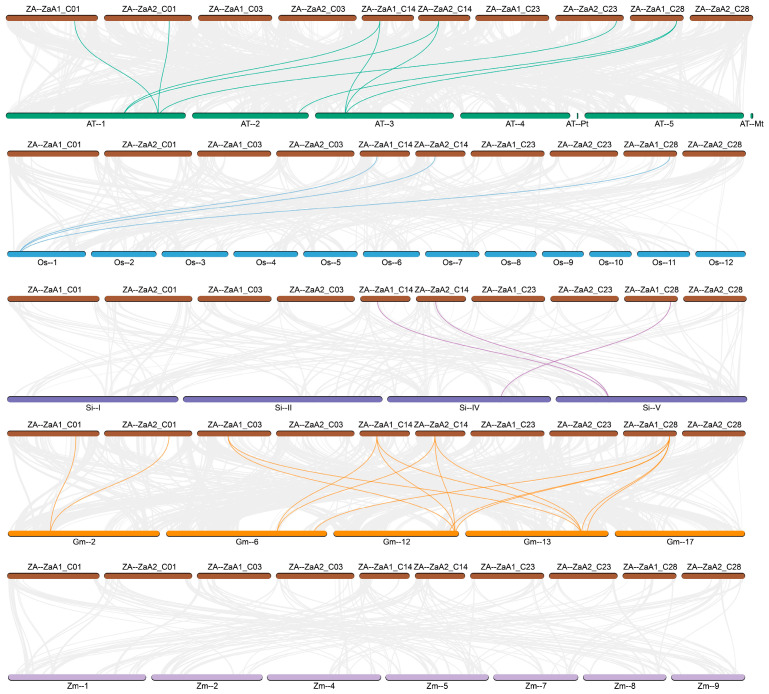
Collinearity analysis of *ZaPEPC* genes with other species. Twelve pairs of *ZaPEPC* genes were analysed for covariance with other species. *Z. armatum* chromosome (ZA–Za) is labelled in brown to distinguish them as the focal species of this study, *A. thaliana* chromosome (At) is marked in green, *O. sativa* chromosome (Os) in blue, *S*. *italica* chromosome (Si) in dark purple, *G. max* chromosome (Gm) in orange, and *Z. mays* chromosome (Zm) in light purple. The grey background is the genomic boundary block between the two species, and the lines |represent homeotic gene pairs with collinear fragments. The analysis reveals that *Z. armatum* shares the most collinear regions with soybean (*G. max*), indicating a closer evolutionary relationship and the retention of more ancestral genomic segments between these two dicot species.

The inter-species collinearity analysis of *ZaPEPC* genes revealed distinct evolutionary trajectories between *Z. armatum* and the five representative plant species (Figure 5). Most notably, *Z. armatum* shared the highest number of collinear regions (15) with soybean. This finding is consistent with their shared evolutionary status as dicots and suggests a high degree of conservation in their PEPC genomic regions. In contrast, fewer collinear regions were detected with the monocot species *O. sativa* (3) and *S. italica* (3), consistent with the greater evolutionary distance and the extensive genome rearrangements known to have occurred since the monocot–dicot divergence. Notably, no collinear regions were identified with *Z. mays*. This absence may be attributed to more genomic rearrangements and variations during evolution. The maize genome underwent a more rapid evolution, including additional whole-genome duplications and chromosomal rearrangements [42]. These results collectively highlight a closer evolutionary relationship between the *PEPC* genes of *Z. armatum* and soybean compared to the monocots, providing a phylogenetic context for future functional studies of these genes (Table S6).

### 3.6. Cis-Elements in the Promoter Regions of ZaPEPC Genes

**Figure 6:**
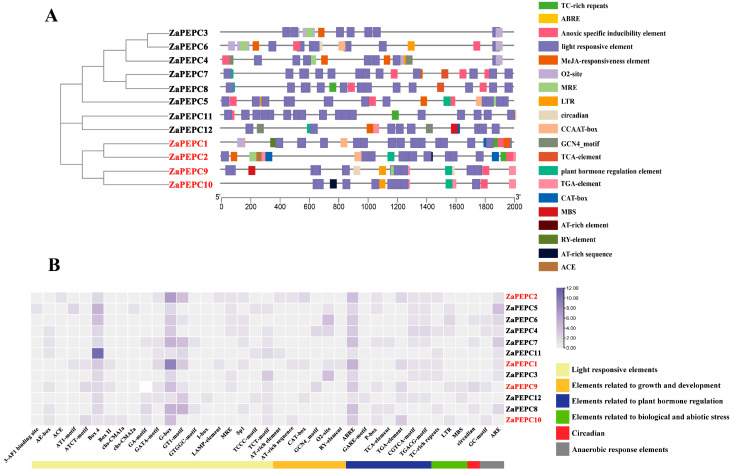
Analysis of *cis*-regulatory elements of the *ZaPEPC* genes. (**A**) Linear analysis of the *cis*-acting elements of the *PEPC* gene family promoter. The red marks represent the bacterial-type PEPC. Different colored boxes represent distinct element types, categorized into four major functions: light responsiveness, phytohormone response (e.g., ABA, MeJA), growth and development, abiotic stress response (e.g., drought, low-temperature) (**B**). Heat map depicting the number of cis-acting elements per promoter. The color scale represents the count of elements for each category in each *ZaPEPC* gene. It maybe suggests the widespread prevalence of these elements across the gene family. The predominance of light-responsive (e.g., G-box) and stress-related elements strongly implies that *ZaPEPC* expression is transcriptionally regulated by environmental factors such as high light intensity and drought.

To study the possible role of *PEPC*s identified in the genome of *Z. armatum* [32], PlantCARE was used to analyze the *cis*-elements in the promoter region of the 2000 bp sequence upstream of the start codon of *ZaPEPC* genes (Figure 6A). These elements were major categorized into four major groups (Figure 6B; Table S7): light responsiveness, phytohormone response, growth and development, abiotic stress response. Promoter analysis revealed an abundance of light-responsive elements. Notably, the G-box was the most prevalent type (*n* = 46). We also identified 20 anaerobic-inducible elements, including GC-motif and ARE (Anaerobic Response Element). All ZaPEPC promoters contained elements responsive to abscisic acid (ABA) and methyl jasmonate (MeJA). Other phytohormone-related elements, such as those for gibberellin (GA), salicylic acid (SA), and auxin, were also detected, suggesting that *ZaPEPC* genes may integrate light and hormonal signals to coordinate photosynthesis and abiotic stress adaptation. Multiple stress-related elements were identified, including TC-rich repeats (defense and stress), LTR (low-temperature), and MBS (drought). Furthermore, elements linked to growth and development were present, such as the O2-site (found in *ZaPEPC3*, *ZaPEPC4*, *ZaPEPC5*, *ZaPEPC6*), CAT-box (meristem expression, in *ZaPEPC1*, *ZaPEPC2*, *ZaPEPC12*), GCN4_motif (endosperm expression, in *ZaPEPC4*, *ZaPEPC9*), RY-element (seed-specific, *ZaPEPC1*), and circadian regulatory elements (*ZaPEPC6*, *ZaPEPC7*, *ZaPEPC8*, *ZaPEPC9*).

### 3.7. Expression Patterns of the PEPC Family in Z. armatum from Three Different Latitudinal Regions

**Figure 7:**
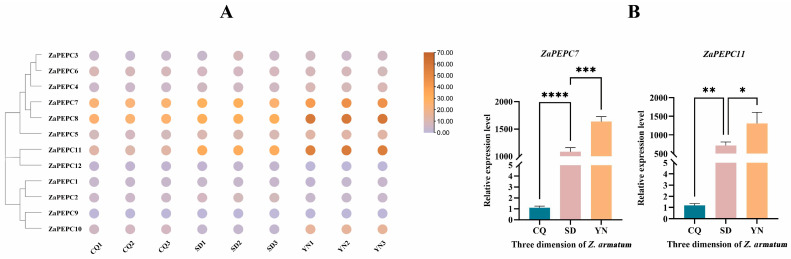
Expression pattern and RT-qPCR analysis of *PEPC* genes at three different latitudinal regions of *Z. armatum*. (**A**) Heatmap of the expression levels of 12 *ZaPEPC* genes across nine individual samples (three biological replicates each from three different regions: Shandong, SD; Chongqing, CQ; Yunnan, YN). Expression values for each gene in each sample are represented as FPKM (Fragments Per Kilobase of transcript per Million mapped fragments) (**B**) The relative expression level of *ZaPEPC7* and *ZaPEPC11* genes at three different latitudes. Error bars represent the mean ± SD of three biological replicates. Asterisks (*) indicate statistical significance of the relative expression level of *Z. armatum* in different latitudes (* *p* < 0.05, ** *p* < 0.01, *** *p* < 0.001, **** *p* < 0.0001).

To analyze the expression patterns of the *ZaPEPC1–12* genes in leaves, we utilized RNA-Seq data and calculated the corresponding FPKM values (Figure 7). The heat map shows the differential expression patterns of the 12 *ZaPEPC* genes in *Z. armatum* at three latitudes in China (Shandong Province, SD; Chongqing Province, CQ; and Yunnan Province, YN) (Figure 7A). Except for *ZaPEPC9* and *ZaPEPC12* from the YN group, which had FPKM values < 1, all other genes exhibited FPKM values > 1 (Table S8), demonstrating the expression of these genes. Notably, *ZaPEPC7* and *ZaPEPC11*—whose promoters are predicted to be enriched in light-responsive cis-elements—showed significantly higher expression in the YN group, consistent with the high-light conditions of this region. We then validated these patterns by RT-qPCR. The results of RT-qPCR showed that the relative expression levels of *ZaPEPC7* and *ZaPEPC11* genes at the three different latitudes were consistent with the transcriptome data (Figure 7B), among which the expression level in the YN group was the highest, followed by the SD group, and the CQ group was the lowest. This strong correlation between in the silico prediction of light responsiveness and the observed environment-specific expression provided experimental support for the proposed regulatory model. The high light intensity and unique climatic conditions in the YN region (a high-altitude tropical area) may activate the light-responsive and stress-responsive elements in the *ZaPEPC7/11* promoters, thereby inducing their high expression levels. This mechanism likely represents a molecular strategy employed by *Z. armatum* to adapt to high-latitude environments.

### 3.8. Kyoto Encyclopedia of Genes and Genomes (KEGG) and Co-Expression Relationship Network Analysis of ZaPEPC Genes

**Figure 8:**
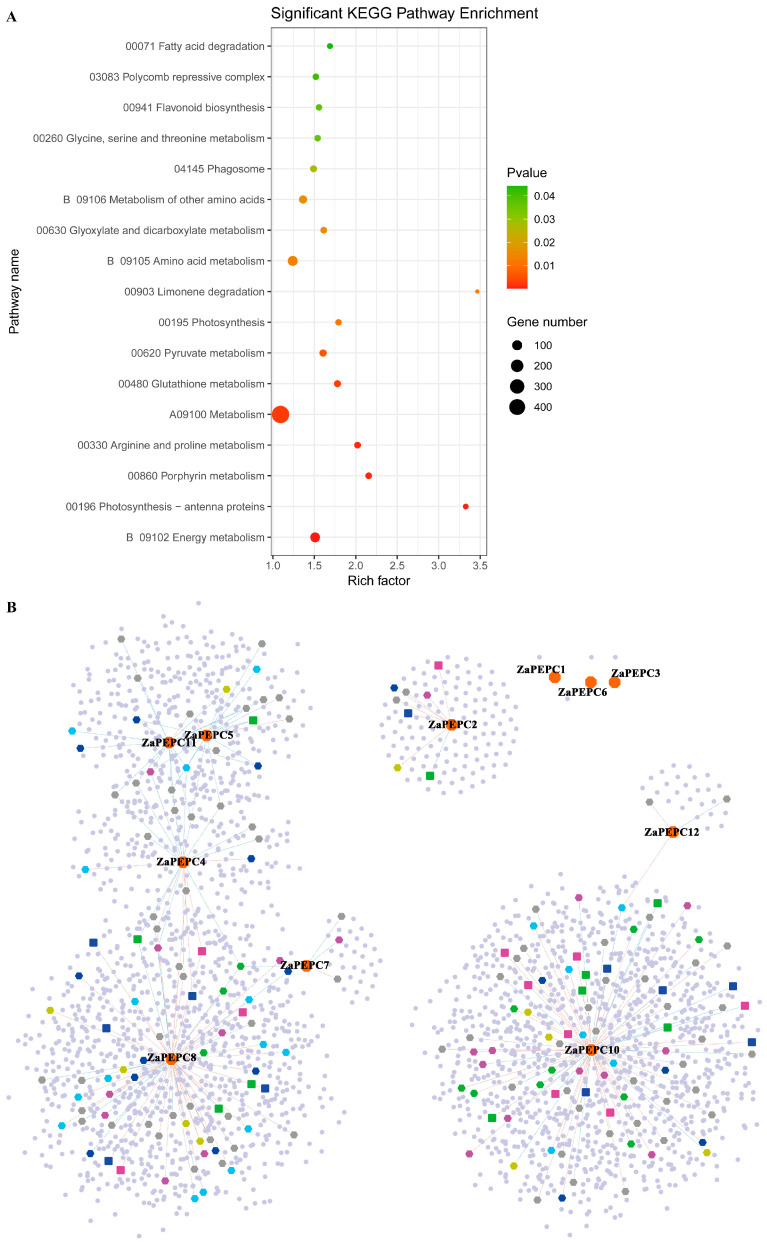
Co-expression relationship network of *ZaPEPC* genes. (**A**) Scatter plot of significant KEGG pathway enrichment. The *X*-axis represents the Rich factor, where a larger value indicates a greater degree of enrichment. It represents the proportion of differentially expressed genes in pathway and proportion of background genes in pathway. The size of the bubble indicates the number of genes enriched in the pathway, and the color represents the significance level (*p*-value). Pathways related to photosynthesis, energy metabolism, and stress responses (e.g., glutathione metabolism) are significantly enriched. (**B**) Co-expression relationship network diagram. Node shapes represent gene categories: orange octagon is *ZaPEPC* gene, purple hexagon is bHLH, blue hexagon is MYB-related, light blue hexagon is WRKY, green hexagon is C_3_H, gold hexagon is ERF, purple square is NAC, blue square is C_2_H_2_, green square is HD-ZIP, gray hexagon is other TFs, and circular blue dots are other genes. Edges (lines) represent significant co-expression relationships between genes, defined by a Pearson correlation coefficient (R) with an absolute value greater than 0.9 and a *p*-value ≤ 0.05. R measures the strength and direction of the linear relationship between the expression profiles of two genes. Red and blue lines denote positive (co-regulated) and negative (inversely regulated) correlations, respectively.

To elucidate the potential biological functions and regulatory networks associated with the *ZaPEPC* genes, we performed a comprehensive co-expression and pathway enrichment analysis. The co-expression network analysis identified a total of 3746 significant co-expressed gene pairs, including 223 transcription factors (Table S9). KEGG pathway enrichment analysis was performed this gene set of 3746 genes and revealed significant associations with photosynthesis and stress-related processes (Figure 8A). Specifically, the pathways for photosynthesis (map00195), photosynthesis–antenna proteins (map00196), and glutathione metabolism (map00480) were significantly enriched. The photosynthesis–antenna proteins pathway showed the highest enrichment factor (approximately 3.5). This implies that certain *ZaPEPC* genes may enhance light energy capture and utilization efficiency while reducing photodamage under high-light conditions. Concurrently, significant enrichment was observed in the core photosynthesis pathway, which provides essential carbon skeletons for secondary metabolism, and in glutathione metabolism (*p*-value = 0.01), indicating a potential role in oxidative stress responses.

The co-expression network further elucidated how these functional associations might be regulated (Figure 8B). Eight *ZaPEPC* genes (*ZaPEPC2*, *ZaPEPC4*, *ZaPEPC5*, *ZaPEPC7*, *ZaPEPC8*, *ZaPEPC10*, *ZaPEPC11*, *ZaPEPC12*) was associated with photosynthesis and stress response related regulatory factors (bHLH, MYB-related, WRKY, C_3_H, ERF, NAC, C_2_H_2_, HD-ZIP) (Figure 8B). Among them, *ZaPEPC4* interacts most frequently with other genes, and interacts with *ZaPEPC11*, *ZaPEPC5*, *ZaPEPC8*, *ZaPEPC7*, respectively. *ZaPEPC5* and *ZaPEPC11* interact most closely with each other, and they are mostly positively correlated with TFs. *ZaPEPC10* showed the highest number of TF connections, primarily positive correlations, followed by *ZaPEPC8*. In contrast, *ZaPEPC2* and *ZaPEPC12* had minimal network interactions (Table S9).

## 4. Discussion

### 4.1. Genome-Wide Identification and Evolutionary Analysis of the PEPC Family in Z. armatum

*Z. armatum* is an economically important crop with significant medicinal value [1]. This study conducted a genome-wide identification of the *ZaPEPC* gene family and analyzed its expression differences across three regions (Shandong, Yunnan, and Chongqing province) to elucidate its potential role in adversity resistance. This research holds great importance for the conservation and utilization of *Z. armatum* germplasm resources. We identified 12 *PEPC* genes in *Z. armatum*, and the number of *ZaPEPC* genes was significantly higher than that of *PEPC* members in foxtail millet (6 *PEPC* genes) [24], *A. thaliana* (4 *PEPC* genes) [22], rice (6 *PEPC* genes) [23], maize (6 *PEPC* genes) [43]. This expansion can likely be attributed to the two ancient polyploidization events documented in *Z. armatum* [32], which often lead to gene family duplication. Previous studies have found that *PEPC* members are divided into two subfamilies: PTPC and BTPC [22]. In this study, eight *ZaPEPC*s were clustered in subfamily I (PTPC) and four were clustered in subfamily II (BTPC). Except for *Z. armatum* (4) and soybean (3), there was only one BTPC in each species (Table S5). The phylogenetic tree showed that *Z. armatum* clusters closely with soybean within the dicot-specific clades, which are distinct from the monocot clades including rice. This clear phylogenetic separation indicates that the *PEPC* gene family diversified before the monocot–dicot split, an event estimated to have occurred around 200 million years ago [44]. The presence of the bacterial-type *PEPC* (BTPC) in both lineages further supports this ancient origin and the hypothesis of a nuclear ancestry for the *PEPC* family [45]. Consequently, this nuclear-derived *PEPC* gene family has gradually differentiated during the evolution of plants, forming a variety of isozymes that adapt to different physiological functions and metabolic needs, thus playing an important role in photosynthesis, stress response, and metabolic regulation in plants [46]. Moreover, collinearity analysis indicated that *PEPC* gene family members showed certain functional conservation during evolution, highlighting that similar biological functions may exist between species [47].

### 4.2. Structural Features and Duplication Events of ZaPEPCs

The composition of introns and exons is closely linked to the evolutionary history of gene families and is often regarded as an evolutionary relic [48]. Our analysis of the gene structure of *ZaPEPCs* revealed clear distinctions between plant-type (PTPC) and bacterial-type (BTPC) members, consistent with patterns reported in other species [49]. Specifically, *ZaPTPC* genes contain 8–10 introns and 9–11 exons, whereas bacterial-type *ZaBTPC* genes possess 19 introns and 20 exons, indicating that the *PEPC* gene family in *Z. armatum* has undergone an evolutionary trajectory similar to that in other plants. In plant evolution, genes often favor shorter introns or even intron loss, which facilitates rapid transcription and enables quicker responses to external environmental stresses [50]. In this context, *ZaPEPC3/4/6/7*, with their relatively compact gene structures, are likely transcribed more rapidly than *ZaPEPC1/2/9/10*, which exhibit more complex architectures. On the other hand, larger introns can promote genetic diversity and functional evolution by providing more sites for alternative splicing and sequence variation [51]. The comparatively long introns in *ZaPEPC1/2/9/10* may thus offer greater potential for structural diversification of PEPC, potentially enhancing the adaptive capacity of *Z. armatum* to environmental challenges over evolutionary time.

Furthermore, systematic analysis revealed distinct correlations between gene structures, protein domain architectures, and the phylogenetic distribution of *ZaPEPCs*. In plants, exon–intron organization is closely associated with alternative splicing, a mechanism that generates diverse transcript isoforms and protein variants, thereby increasing functional diversity and facilitating rapid adaptation to environmental changes [52]. In this study, we found that the plant-type *ZaPEPCs* (PTPCs; including *ZaPEPC3/4/5/6/7/8/11/12*) typically contain 8–10 introns and 9–11 exons, and encode proteins with 10 conserved motifs arranged in a highly consistent order. This relatively simple and conserved gene architecture may facilitate rapid transcription and translation [52], potentially enabling *Z. armatum* to efficiently respond to common environmental fluctuations. For instance, in high-altitude, high-light regions such as Yunnan, plant-type PTPCs may help enhance photosynthetic efficiency and support basic stress acclimation [16]. In contrast, four bacterial-type *ZaPEPCs* (BTPCs; *ZaPEPC1/2/9/10*) exhibit more complex gene structures, with 19 introns and 20 exons, along with a distinct arrangement of conserved motifs—most notably, a shifted position of Motif10. This structural complexity and domain reorganization suggest that BTPCs may play a role in adapting to more severe or sustained environmental stresses. Under extensive stress conditions, complex gene structures can promote alternative splicing, enabling these genes to acquire specialized metabolic functions and improve evolutionary adaptability [53,54]. In summary, the *ZaPEPC* gene family exhibits coordinated evolution of gene and protein structures. The two subfamilies, BTPC and PTPC, have evolved distinct structural and functional characteristics, working in concert to enhance the resilience of *Z. armatum* to both common and extreme environmental challenges.

Gene duplication events are fundamental drivers of gene family expansion and functional diversification [55]. These events are broadly classified into two categories: whole-genome duplication (WGD), which can lead to genome doubling and species differentiation, and regional duplications (such as segmental or tandem duplications), which primarily contribute to the expansion of specific gene families [56]. The PEPC gene family in *Z. armatum* has undergone notable expansion, with its member count being two to three times that of rice, *A. thaliana*, maize, and foxtail millet. This observation suggests that *Z. armatum* may have experienced one or more whole-genome duplication events. Synteny analysis further classified the *ZaPEPC* genes into two major segmental duplication groups: one comprising *ZaPEPC1*/2/910 (bacterial-type, BTPC), and the other consisting of *ZaPEPC3*/5/7/8 (plant-type, PTPC). This grouping indicates that BTPC and PTPC represent two distinct evolutionary branches that emerged during the expansion of the *PEPC* gene family in this species. To understand the evolutionary pressures acting on these genes, we calculated the Ka/Ks ratios for nine duplicated gene pairs. All pairs showed values greater than 0 but less than 1 (Table 3), indicating that they have undergone strong purifying selection. This pattern, which is consistent with observations in other plant species such as foxtail millet and bamboo [24,47], underscores the essential and evolutionarily conserved role of the PEPC enzyme in plant metabolism.

### 4.3. Regulatory Mechanisms and Expression Patterns of ZaPEPCs

To elucidate the transcriptional regulation of *ZaPEPC* genes, we performed a systematic analysis of predicted cis-acting elements in their promoter regions. Studies have shown that genes involved in multiple stress responses are often closely related to cis-acting elements in their promoter region [57]. The primary function of cis-regulatory elements in gene upstream regions is to serve as transcription factor (TF) binding sites, enabling their crucial role in controlling gene expression [58]. Promoter analysis revealed an abundance of putative light-responsive elements, consistent with a potential role for light in regulating PEPC function. More importantly, the promoter contains multiple predicted hormone-responsive elements, especially ABA and MeJA. This suggests that *ZaPEPC* expression might be induced by ABA and MeJA, potentially integrating these signals into the plant’s stress response and signaling pathways [59]. The presence of these predicted elements also implies a potential role in helping plants cope with environmental challenges such as drought [60]. Gibberellin (GA), a phytohormone known to regulate processes such as cell division, seed germination, and fruit development [61], may exert its effects through specific downstream components. Notably, the identification of predicted GA-responsive elements suggests that *PEPC* could be one such component within the GA signaling pathway, which coordinately regulates plant growth and stress resistance [62]. Although *PEPC* genes exhibit a high degree of conservation, this conserved gene family performs diverse functions, including photosynthetic and non-photosynthetic functions, for survival in terrestrial environments [13]. The diverse predicted cis-regulatory landscape of the *ZaPEPC* family offers valuable insights into how hormones and environmental cues modulate its activity. These results advance our understanding of the intricate control mechanisms governing plant adaptation, with implications for engineering stress-resilient crops.

KEGG (Kyoto Encyclopedia of Genes and Genomes) pathway enrichment analysis indicated that the *ZaPEPC* genes are predominantly enriched in pathways related to photosynthesis and stress-related metabolism. Notably, arginine and proline metabolism (map00330) and glutathione metabolism (map00480), both critical for osmotic adjustment and oxidative defense, respectively [63,64], were significantly enriched. As a key anaplerotic enzyme, PEPC replenishes the tricarboxylic acid (TCA) cycle by catalyzing the conversion of phosphoenolpyruvate (PEP) to oxaloacetate (OAA), thereby supplying essential carbon skeletons for biosynthesis and energy metabolism. It is noteworthy that intermediates from arginine and proline metabolism (e.g., glutamate) directly feed into the TCA cycle, thereby linking carbon and nitrogen metabolism [65]. Thus, under favorable growth conditions such as those in Yunnan, high *ZaPEPC* expression may bolster the cellular pool of carbon skeletons, priming the plant for rapid synthesis of osmolytes like proline upon stress onset [66]. Furthermore, co-expression network analysis revealed that eight *ZaPEPC* genes exhibit positive or negative correlations with eight transcription factors (TFs), including bHLH, MYB-related, WRKY, C_3_H, ERF, NAC, C_2_H_2_, and HD-ZIP. Most of these TFs are well-documented regulators of environmental stress responses [67,68,69]. For instance, in *Iris laevigata*, IlWRKY22 enhances total chlorophyll content, reduces stomatal aperture, boosts antioxidant enzyme activities, and improves photosynthetic efficiency under stress. Similarly, NAC TFs help maintain photosynthetic performance and modulate hormone signaling during drought [67]. Additionally, certain *bHLH* members mediate light signaling pathways [69]. These co-expression relationships suggest that *ZaPEPCs* operate within a coordinated regulatory network, reinforcing the functional link between photosynthetic carbon metabolism and stress adaptation.

Our expression analysis revealed a distinct spatial pattern for *ZaPEPC7* and *ZaPEPC11*, with transcript levels highest in Yunnan (YN), intermediate in Shandong (SD), and lowest in Chongqing (CQ). This pattern, consistently observed in both RNA-seq and qRT-PCR data, correlates with the contrasting environmental conditions across these regions: YN (plateau, temperate climate, ample light and rainfall) > SD (high-latitude, cold-dry, high light intensity) > CQ (lowland, humid-hot, low light). Phylogenetically, *ZaPEPC7* clusters closely with soybean genes *GmPEPC6*, *GmPEPC8*, and *GmPEPC9*, which were established regulators of diverse abiotic stresses, including aluminum toxicity, chilling, and salinity [16]. This strong phylogenetic conservation suggests that *ZaPEPC7* may fulfills a similar role in stress adaptation in *Z. armatum*. The significant variation in PEPC gene expression across latitudes implies a substantial influence of environmental factors such as light, temperature, and humidity. This regulatory potential is further supported by the presence of numerous light-, hormone-, and stress-responsive *cis*-elements in the promoters of *Z. armatum* PEPC genes. We hypothesize that in the high-light environment of Yunnan, light-signaling pathways activate specific transcription factors that bind to these promoter elements, thereby initiating the elevated expression of *ZaPEPC7* and *ZaPEPC11* [70]. This hypothesis is reinforced by our co-expression network analysis, which revealed significant correlations between *ZaPEPC*s and stress-associated transcription factors (e.g., NAC, WRKY). These TFs may constitute a regulatory module that coordinately controls *PEPC* expression alongside other stress-responsive genes, forming an integrated adaptive network. Moreover, light is a key inducer of PEPC kinase (PPCK) expression and activity [71]. PPCK phosphorylates PEPC at a conserved serine residue, alleviating feedback inhibition by malate and thereby enhancing enzyme activity [26,72]. As a central enzyme in plant carbon metabolism, the differential expression of PEPC across latitudes underscores its role in modulating carbon metabolic strategies in *Z. armatum* in response to environmental gradients. This study establishes a foundation for elucidating the regulatory mechanisms of *ZaPEPC7* and *ZaPEPC11*. Future work should employ functional genetic tools, such as the CRISPR-Cas9 system [73] and RNA interference (RNAi) [74], to precisely manipulate these genes and investigate their molecular functions. Such insights will be of considerable theoretical importance for developing new, superior cultivars of Sichuan pepper with enhanced stress resilience.

## 5. Conclusions

In summary, this study presents a systematic identification and characterization of 12 *ZaPEPC* genes in *Z. armatum*, revealing their structural conservation, evolutionary divergence, and potential roles in environmental adaptation. The *ZaPEPC* family members are classified into plant-type (PTPC) and bacterial-type (BTPC) subfamilies, which exhibit conserved functional domains and distinct gene structures. Gene duplication events have contributed to the expansion of this gene family. Phylogenetic analysis revealed a closer homology between *ZaPEPC*s and soybean PEPCs, suggesting shared evolutionary origins that predate the monocot–dicot divergence. Promoter *cis*-element analysis identified an abundance of stress-responsive motifs, such as those associated with light, ABA, and MeJA, indicating the potential involvement of *ZaPEPC*s in abiotic stress regulation. Expression profiling further demonstrated environment-specific expression patterns, with notably elevated transcript levels of *ZaPEPC7* and *ZaPEPC11* in the Yunnan group, underscoring their likely role in local environmental adaptation. Collectively, these findings provide fundamental insights into the functional diversity and stress response mechanisms of the *ZaPEPC* gene family, and highlight promising targets for improving photosynthetic efficiency and stress tolerance in *Z. armatum*. Future research should prioritize functional validation of key genes such as *ZaPEPC7* and *ZaPEPC11* using reverse genetics tools like CRISPR-Cas9 and RNAi, and further explore their regulatory networks and metabolic contributions under diverse stress conditions to support molecular breeding efforts.

## Figures and Tables

**Table 1:** Basic physical and chemical properties of ZaPEPC proteins.

Gene ID	Rename	Size (Amino Acids)	Molecular Weight (kDa)	Aliphatic Index	Instability Index	Isoelectric Point (pI)	Grand Average of Hydropathicity
ZaA2_C01.Contig2868.3	*ZaPEPC1*	1039	116.85	88.4	50.99	7.33	−0.411
ZaA1_C01.Contig900.7	*ZaPEPC2*	1057	118.67	88.3	51.69	6.15	−0.412
ZaA2_C03.Contig267.3	*ZaPEPC3*	969	110.99	92.12	47.93	6	−0.375
ZaA1_C03.Contig793.20	*ZaPEPC4*	960	109.93	91.76	46.79	5.78	−0.361
ZaA1_C03.Contig1140.18	*ZaPEPC5*	867	99.63	89.67	48.91	6.51	−0.384
ZaA1_C03.Contig2837.3	*ZaPEPC6*	969	110.96	90.41	49.15	6	−0.395
ZaA1_C14.Contig1122.26	*ZaPEPC7*	969	110.82	91.52	46.83	5.96	−0.385
ZaA2_C14.Contig391.47	*ZaPEPC8*	1115	127.93	94.91	49.00	6.45	−0.307
ZaA2_C05.Contig15.2.10	*ZaPEPC9*	1034	115.77	89.69	48.89	6.16	−0.366
ZaA2_C23.Contig1171.3	*ZaPEPC10*	1034	115.71	90.07	49.78	6.12	−0.356
ZaA1_C28.Contig69.318	*ZaPEPC11*	965	110.37	90.25	44.48	5.98	−0.393
ZaA2_C10.Contig6.1.47	*ZaPEPC12*	965	110.27	90.05	44.15	5.90	−0.391

**Table 2:** Secondary structure of PEPC family proteins.

Gene	Alpha Helix (%)	Extended Strand (%)	Beta Turn (%)	Random Coil (%)	Subcellular Localization
*ZaPEPC1*	56.69%	6.06%	4.43%	32.82%	Cytoplasm
*ZaPEPC2*	57.62%	6.62%	4.16%	31.60%	Cytoplasm
*ZaPEPC3*	61.71%	5.88%	3.92%	28.48%	Cytoplasm
*ZaPEPC4*	60.94%	5.94%	4.17%	28.96%	Cytoplasm
*ZaPEPC5*	59.98%	6.46%	4.27%	29.30%	Cytoplasm
*ZaPEPC6*	61.71%	6.19%	4.23%	27.86%	Cytoplasm
*ZaPEPC7*	59.96%	6.19%	4.13%	29.72%	Cytoplasm
*ZaPEPC8*	57.94%	8.79%	4.75%	28.52%	Cytoplasm
*ZaPEPC9*	56.09%	6.19%	4.16%	33.56%	Cytoplasm
*ZaPEPC10*	57.35%	6.00%	3.97%	32.69%	Cytoplasm
*ZaPEPC11*	61.87%	6.01%	4.04%	28.08%	Cytoplasm
*ZaPEPC12*	61.66%	6.22%	4.15%	27.98%	Cytoplasm

**Table 3:** Analysis of duplication events of *PEPC* gene pairs.

Gene 1	Gene 2	Ka	Ks	Ka/Ks
*ZaPEPC9*	*ZaPEPC2*	0.0273	0.1763	0.1550
*ZaPEPC9*	*ZaPEPC10*	0.0025	0.0193	0.1315
*ZaPEPC10*	*ZaPEPC1*	0.0452	0.1996	0.2266
*ZaPEPC10*	*ZaPEPC2*	0.0282	0.1867	0.1511
*ZaPEPC1*	*ZaPEPC2*	0.0174	0.0278	0.6234
*ZaPEPC1*	*ZaPEPC9*	0.0438	0.1907	0.2299
*ZaPEPC7*	*ZaPEPC8*	0.0069	0.0189	0.3656
*ZaPEPC7*	*ZaPEPC4*	0.0176	0.2593	0.0679
*ZaPEPC3*	*ZaPEPC4*	0.0054	0.0167	0.3249

## Data Availability

The original data presented in the study have been deposited in the National Genomics Data Center with the primary accession number PRJCA037036 and are openly available at the following URL: https://ngdc.cncb.ac.cn/gsa/search?searchTerm=PRJCA037036 (accessed on 10 November 2025).

## Supplementary Materials

The following supporting information can be downloaded at: https://www.mdpi.com/article/10.3390/biology14111605/s1, Table S1: RT-qPCR primers for *ZaPEPCs* gene; Table S2: The *PEPC* gene family sequence of six species; Table S3: The information on *ZaPEPCs* and their upstream and downstream contigs; Table S4: Phylogenetic mapping of the *ZaPEPC* gene; Table S5: Distribution of *PEPC* in five different species; Table S6: The collinearity analysis of *ZaPEPC* genes; Table S7: Distribution of regulatory elements of the *ZaPEPC* gene family; Table S8: The FPKM values of 12 *ZaPEPC* genes; Table S9: *ZaPEPC* gene co-expression; Figure S1: Amino acid sequence diagram of the *ZaPEPC* gene family.
