# Supplementary figures and images for: Genome-Wide Identification and Expression Analysis of the PEPC Gene Family in Zanthoxylum armatum Reveals Potential Roles in Environmental Adaptation
Source: Biology (Basel). 2025 Nov 16;14(11):1605. doi: 10.3390/biology14111605 (PMC12650240; doi:10.3390/biology14111605)

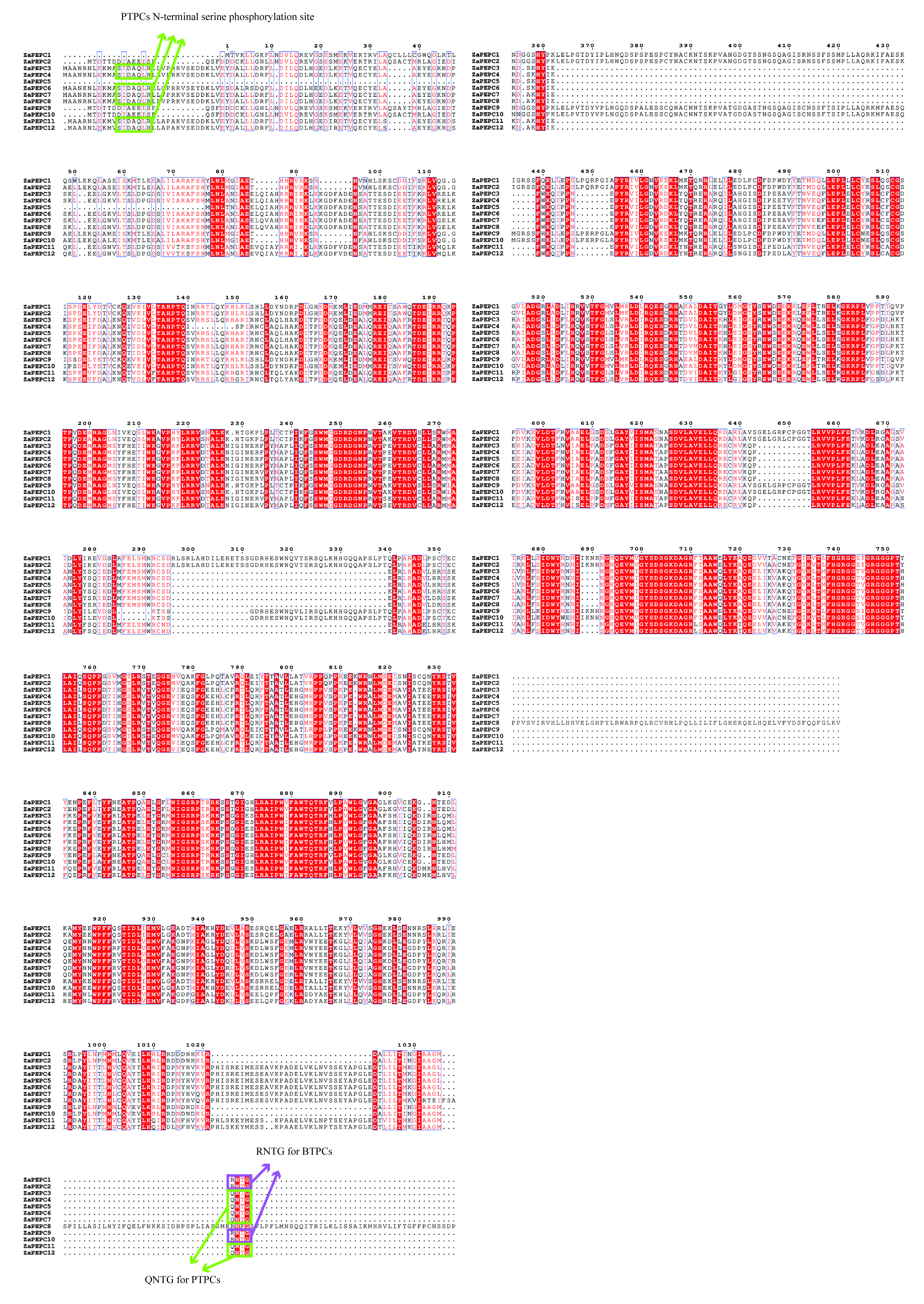

Supplement: Supplementary file 1 [file biology-14-01605-s001.zip › Figure S1 Amino acid sequence diagram of the ZaPEPC gene family.tif]
